# New method of remnant closure during distal pancreatectomy

**DOI:** 10.1007/s00423-023-02916-2

**Published:** 2023-05-04

**Authors:** D. Kelemen, A. Kerbeche, N. Farkas, A. Vereczkei

**Affiliations:** 1 Department of Surgery, Medical Faculty, Clinical Center, University of Pécs, Ifjúság u. 13., 7624 Pécs, Hungary; 2 Institute of Bioanalysis, Medical Faculty, University of Pécs, Honvéd u. 1., 7624 Pécs, Hungary

**Keywords:** Fascia graft, Circular fixation stitch, Pancreatic remnant closure, Distal pancreatectomy, Pancreatic fistula

## Abstract

**Purpose:**

Pancreatic fistula following distal pancreatectomies still remains a relevant problem. The present study describes our first series with a new method of pancreatic remnant closure.

**Methods:**

A free fascia-peritoneum graft — harvested from the internal rectus sheet — was fixed onto the pancreatic stump by one circular stitch. The method was applied in 18 cases.

**Results:**

The postoperative hospital stay was 8 days in average. No clinically relevant postoperative pancreatic fistula (CR-POPF) developed. The morbidity rate was 39%, mostly Clavien-Dindo Grade II types. There was no reoperation or mortality.

**Conclusion:**

The first series showed advantageous results with our method. Certainly, further studies are needed for the evaluation of this new and promising technique.

## Introduction

The frequency of a pancreatic fistula after distal pancreatectomies is around 30% [1]. This complication can be the major source of further morbidity and increased hospital stay. Randomized trials and consensus conferences investigated and compared the different methods for the management of the pancreatic remnant, like stapler vs. hand-sewn closure, teres ligament patch vs. no patch, pancreato-enteric anastomosis vs. hand-sewn closure, administration of somatostatin analogs vs. no administration [2-5]. Most trials did not find any convincing differences among the techniques, concerning their impact on the development of a pancreatic fistula. However, there is an agreement that further trials and novel approaches are needed [5]. The present study describes a new method for the pancreatic remnant closure and the experience with a pilot series. The main purpose was to investigate the influence of our technique on the development of a clinically relevant postoperative pancreatic fistula (CR-POPF), classified by the ISGPS [6].

## Material and methods

Between January 2019 and November 2022, our new method was applied in 18 consecutive cases during open distal pancreatectomies, all performed by a single surgeon. During this period, the well-known methods (fish-mouth closure, teres ligament patch or stapler) were only utilized by other surgeons of the department.

**Fig. 1:**
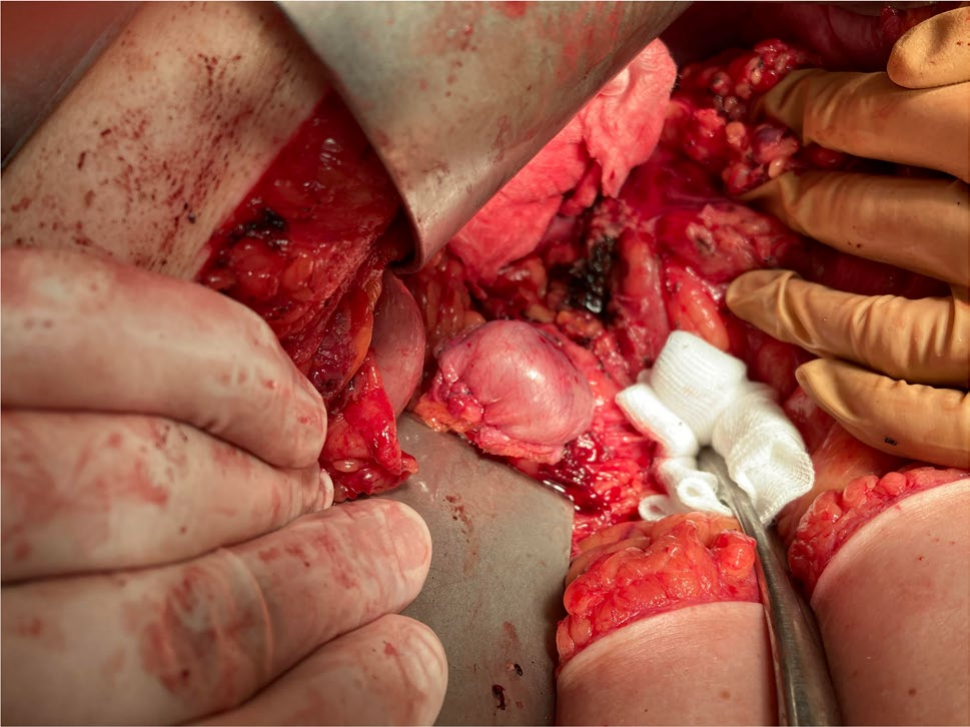
On the operative picture, the pancreatic stump is covered with a fascia-peritoneum graft (in this case the peritoneal surface is outside) and fixed by one circular stitch, knotted tightly

**Fig. 2:**
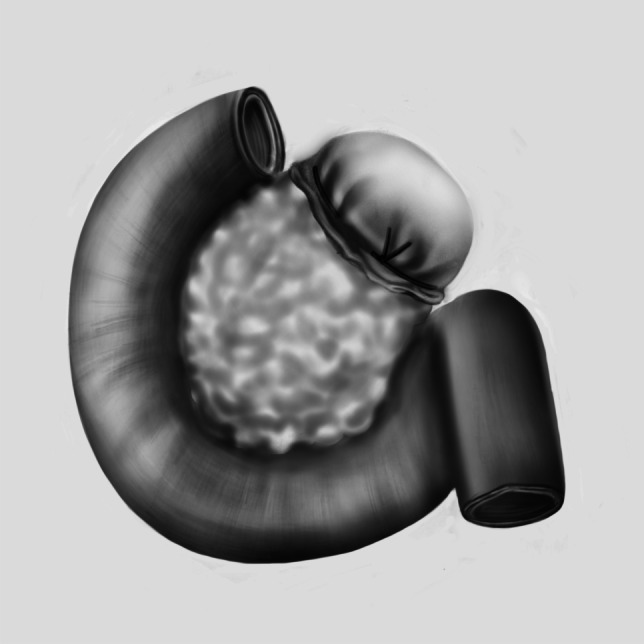
Illustration of the method

In this series the pancreatic neck was transected with a scalpel, and the Wirsung’s duct was closed by a Z-shaped atraumatic non-absorbable stitch in all cases. For the remnant closure, a new method was applied, namely, the pancreatic cut surface was covered with a free fascia-peritoneum graft. Its withdrawal was very easily done by the excision of a patch from the internal part of the rectus sheet at the edge of the subcostal laparotomy wound. This autologous graft certainly contained not only the fascia, but it had also a peritoneal side. However, we did not care about which side (fascial or peritoneal) of the flap covered the cut end. Afterwards, it was fixed by one circular stitch (3/0 monofilament non-absorbable suture). This single suture was stitched into the pancreatic parenchyma generally at four points (three to five, depending on the stump size), cranially, dorsally, caudally, and ventrally. The aim was to create as few stitch holes as possible. The suture line was driven about 1 cm from the transection surface, then it was tightly knotted (shown in Figs. 1 and 2). A soft silicon drain was positioned beside the pancreas, and the amylase level of drain fluid was measured on each postoperative day. The clinically relevant postoperative pancreatic fistula (CR-POPF) was defined, according to the updated classification of the ISGPS [6]. On the first postoperative day, oral feeding was started. Perioperative thrombosis and antibiotic prophylaxis was applied. Written informed consent was obtained from the patients.

## Results

Table 1 shows the pre- and early postoperative data. The ratio of female patients was much higher than the male one. The mean age was 59 years (range: 36–74). Most of the patients were in ASA Class. II. Surgery in these cases was indicated mainly for pancreatic tumors (9 cancers, 5 endocrine neoplasms, moreover three cystic tumors) and in a single patient for gastric cancer. In malignant cases, splenectomy and regional lymphadenectomy were routine measures. Besides the pancreatic resection, additional procedures were also performed, namely, three cholecystectomies, two left adrenalectomies, two colon resections, one liver metastasectomy, and one total gastrectomy. Duration of the operations was 165 min in average (range: 125–350). During four procedures, 2 units transfusion was needed due to the blood loss. The postoperative hospital stay was 8 days in average (range: 6–15). The peripancreatic drain was left in place for 6 days in average, until the amylase value decreased or remained under 1000 U/l. Early morbidity occurred in 7 patients (39%), mainly Grade II according to the Clavien-Dindo classification. There was no CR-POPF, reoperation, or mortality.

**Table 1:** Pre- and early postoperative data (*n*: 18)

Gender M/F	5/13
Age (y) mean (range)	59 (36–74)
ASA	
Class I	4
Class II	12
Class III	2
Duration of operation (min.) mean (range)	165 (125–350)
Need of transfusion (unit) mean (range)	0.4 (0–2)
Hospital stay (d) mean (range)	8 (6–15)
Morbidity (*n*/%)	7 (39%)
Segmental pulmonary embolism	1
Uroinfection	1
Wound healing disorder	1
Transient atrial fibrillation	1
Anemia	3
Clavien-Dindo classification (*n*)	
Grade I	1
Grade II	6
CR-POPF (*n*/%)	0
Reoperation, mortality	0
Diagnosis	
Pancreatic cancer	9
Pancreatic endocrine tumor	5
Pancreatic cystic tumor	3
Gastric cancer	1

## Discussion

Despite many efforts and innovations, there has been no convincing progression in the reduction and prevention of the pancreatic fistula rate after distal pancreatectomies. There are still some open questions. Regarding the steps of the procedure, the first is how to divide the pancreas? In case of a scalpel transection, it is easy to identify and ligate the Wirsung’s duct separately, which is recommended even in the lack of a randomized trial [7]. However, this transection method requires a covering [8]. Other options are the different tissue sealing devices, like mono- or bipolar electrocautery, ultrasonic scalpel, or thermofusion device. These instruments not only divide the pancreas, but at the same time coagulate the cut surface. Even after their combination with biologic sealants, like TachoSil, the postoperative pancreatic fistula rate has not decreased [5]. A randomized controlled trial of stapled versus ultrasonic transection demonstrated no significant difference in the rate of postoperative pancreatic fistula [9]. After the pancreatic transection, the next step is to close the remnant surface. The traditional method is the hand-sewn one. The cut surface is excised in a “fish-mouth” fashion, and then it is closed with single or continuous stitches. In the laparoscopic era, the stapler closure technique became obviously popular; however, this method, compared to the traditional hand-sewn one [2], and even the reinforced staple-line technique, compared to the standard stapler method [10], had no impact on the pancreatic fistula rate. A further option to cover the closed surface is the use of autologous tissues, like the teres ligament flap. The utilization of this technique decreased the overall complication rate, but not the occurrence of CR-POPF [3]. Similar results were found after covering the staple-line with a seromuscular jejunal patch [11]. Randomized studies investigated the use of a pancreato-enteric anastomosis; however, this also did not decrease the pancreatic fistula rate [4, 12]. As a promising option, the preoperative endoscopic botulinum toxin injection into the sphincter of Oddi has been introduced, according to the preliminary results [13]. As there is no consensus regarding the routine abdominal drainage, the prophylactic use of somatostatin analogs, and the pancreatic duct stenting, further randomized multicenter trials and novel approaches are needed [5].

Autologous fascia graft — like fascia lata — is widely used by the orthopedic and general surgeons, because it can be transferred freely and safely; moreover, it becomes incorporated into the host tissues and preserves its function even in case of a bacterial contamination [14, 15]. In our method, the autograft could conveniently be excised from the inner rectus sheet in an adequate size, in order to cover the pancreatic stump. It was strong and resistant against the pancreatic enzymes, as the juice leaking through the transection surface contains only the lipase in an active form, which is less harmful for the tissue of the fascia. On the pancreatic stump, there is no peritoneum, so for the covering, it was not important to use the peritoneal side of the graft, while the advantageous healing process of contiguous serosal surfaces — like in case of a bowel anastomosis — cannot be achieved during pancreatic remnant closures. The edges of the cut surface were not sutured together, but the Wirsung’s duct was closed with a Z-shaped stitch. It is an interesting question, whether the hand-sewn or stapler closure can add further protection in the prevention of CR-POPF. However the available data in the literature does not support any additive value of the stapled transection, not even with reinforcement.

The circular stitch (3/0 non-absorbable thread) was tightly knotted; however, the strong constitution of the flap prevented the burst of the pancreatic parenchyma by the suture. Stitches in a minimal number were placed to decrease the deleterious effect of the stitch holes, as these channels may facilitate the leak of the pancreatic juice and subsequently the development of a fistula. After tight fixation of the graft onto the stump, the pancreatic juice was hardly capable to accumulate and leak, due to the lack of space and also due to the compression of small ducts by the tight knot. Regarding the drain management, our policy may be changed in the future, due to two reasons. First, we have not detected any pancreatic fistula in our series. Furthermore, new observations have emerged, namely, a recently published retrospective multicenter study found lower rate of CR-POPF, if the peripancreatic drainage was selectively omitted during distal pancreatectomy. It was also established that multicenter randomized trials are needed to confirm the safety of this selective no-drain strategy and to define subgroups of patients, who may rather benefit from drainage [16].

## Conclusion

In this study, a new method of remnant closure was investigated during open distal pancreatectomies, namely, the pancreatic cut surface was covered with a free fascia-peritoneum graft — harvested from the inner rectus sheet — which was fixed by one circular stitch. CR-POPF did not develop after this technique in any case. However, more cases and a comparison to other methods are needed to prove its benefit.

## Data Availability

The data that support the findings of this study are not publicly available, because their containing information could compromise the privacy of the participants, but are available from the corresponding author (Dezső Kelemen). Further enquiries can be directed to the corresponding author.

## References

[1] Harnoss JC, Ulrich AB, Harnoss JM, Diener M, Büchler MW, Weisch T (2014). Use and results of consensus definitions in pancreatic surgery: a systematic review. Surgery.

[2] Diener MK, Seiler CM, Rossion I, Kleeff J, Glanemann M, Butturini G (2011). Efficacy of stapler versus hand-sewn closure after distal pancreatectomy (DISPACT): a randomized, controlled multicentre trial. Lancet.

[3] Hassenpflug M, Hinz U, Strobel O, Volpert J, Knebel P, Diener M (2016). Teres ligament patch reduces relevant morbidity after distal pancreatectomy (the DISCOVER Randomized Controlled Trial). Ann Surg.

[4] Kawai M, Hirono S, Okada K, Sho M, Nakajima Y, Eguchi H (2016). Randomized controlled trial of panreaticojejunostomy versus stapler closure of the pancreatic stump during distal pancreatectomy to reduce pancreatic fistula. Ann Surg.

[5] Miao Y, Lu Z, Yeo C, Vollmer C, Fernandez-del Castillo C, Ghaneh P, International Study Group of Pancreatic Surgery (ISGPS) (2020). Management of the pancreatic transection plane after left (distal) pancreatectomy: expert consensus guidelines by the International Study Group of Pancreatic Surgery (ISGPS). Surgery.

[6] Bassi C, Marchegiani G, Dervenis C, Sarr M, Abu Hilal M, Adham M, International Study Group of Pancreatic Surgery (ISGPS) (2017). The 2016 update of the International Study Group (ISGPS) definition and grading of postoperative pancreatic fistula: 11 years after. Surgery.

[7] Bilimoria MM, Cormier JN, Mun Y, Lee JE, Evans DB, Pisters PW (2003). Pancreatic leak after left pancreatectomy is reduced following main pancreatic duct ligation. Br J Surg.

[8] Kollár D, Huszár T, Pohárnok Z, Cselovszky É, Oláh A (2016). A review of techniques for closure of the pancreatic remnant following distal pancreatectomy. Dig Surg.

[9] Landoli L, De Pastena M, Fontan M, Malleo G, Esposito A, Casetti L (2021). A randomized controlled trial of stapled versus ultrasonic transection in distal pancreatectomy. Surg Endosc.

[10] Wennerblom J, Ateeb Z, Jönsson C, Björnsson B, Tingstedt B, Williamsson C (2021). Reinforced versus standard stapler transection on postoperative pancreatic fistula in distal pancreatectomy: multicentre randomized clinical trial. Br J Surg.

[11] Oláh A, Issekutz A, Belágyi T, Hajdu N, Romics I (2009). Randomized clinical trial of techniques for closure of the pancreatic remnant following distal pancreatectomy. Br J Surg.

[12] Uemura K, Satoi S, Motoi F, Kwon M, Unno M, Murakami Y (2017). Randomized clinical trial of duct-to-mucosa pancreaticogastrostomy versus handsewn closure after distal pancreatectomy. Br J Surg.

[13] Hackert T, Klaiber U, Hinz U, Kehayova T, Probst P, Knebel P (2017). Sphincter of Oddi botulinum toxin injection to prevent pancreatic fistula after distal pancreatectomy. Surgery.

[14] Kartus J, Movin T, Karlsson J (2001). Donor-site morbidity and anterior knee problems after anterior cruciate ligament reconstruction using autograft. Arthroscopy.

[15] Sekine Y, Sugo H, Iwanaga N, Neshime S, Watanoba I (2020) Relaparotomy two years after incisional hernia repair using a free fascia lata graft. Case Rep Sur (1769404):4. 10.1155/2020/17694041 10.1155/2020/1769404 PMC7093902 32231844

[16] van Bodegraven EA, De Pastena M, Vissers FL, Balduzzi A, Stauffer J, Esposito A (2022). Routine prophylactic abdominal drainage versus no-drain strategy after distal pancreatectomy: a multicenter propensity score matched analysis. Pancreatology.

